# Detecting *In Situ* Copepod Diet Diversity Using Molecular Technique: Development of a Copepod/Symbiotic Ciliate-Excluding Eukaryote-Inclusive PCR Protocol

**DOI:** 10.1371/journal.pone.0103528

**Published:** 2014-07-24

**Authors:** Simin Hu, Zhiling Guo, Tao Li, Edward J. Carpenter, Sheng Liu, Senjie Lin

**Affiliations:** 1 Key Laboratory of Tropical Marine Bio-resources and Ecology, South China Sea Institute of Oceanology, Chinese Academy of Sciences, Guangzhou, China; 2 University of Chinese Academy of Sciences, Beijing, China; 3 Tropical Marine Biological Research Station in Hainan, Chinese Academy of Sciences, Sanya, China; 4 Romberg Tiburon Center, San Francisco State University, San Francisco, California, United States of America; 5 Marine Biodiversity and Global Change Research Center, Xiamen University, Xiamen, China; 6 Department of Marine Sciences, University of Connecticut, Groton, Connecticut, United States of America; Dauphin Island Sea Lab, United States of America

## Abstract

Knowledge of in situ copepod diet diversity is crucial for accurately describing pelagic food web structure but is challenging to achieve due to lack of an easily applicable methodology. To enable analysis with whole copepod-derived DNAs, we developed a copepod-excluding 18S rDNA-based PCR protocol. Although it is effective in depressing amplification of copepod 18S rDNA, its applicability to detect diverse eukaryotes in both mono- and mixed-species has not been demonstrated. Besides, the protocol suffers from the problem that sequences from symbiotic ciliates are overrepresented in the retrieved 18S rDNA libraries. In this study, we designed a blocking primer to make a combined primer set (copepod/symbiotic ciliate-excluding eukaryote-common: CEEC) to depress PCR amplification of symbiotic ciliate sequences while maximizing the range of eukaryotes amplified. We firstly examined the specificity and efficacy of CEEC by PCR-amplifying DNAs from 16 copepod species, 37 representative organisms that are potential prey of copepods and a natural microplankton sample, and then evaluated the efficiency in reconstructing diet composition by detecting the food of both lab-reared and field-collected copepods. Our results showed that the CEEC primer set can successfully amplify 18S rDNA from a wide range of isolated species and mixed-species samples while depressing amplification of that from copepod and targeted symbiotic ciliate, indicating the universality of CEEC in specifically detecting prey of copepods. All the predetermined food offered to copepods in the laboratory were successfully retrieved, suggesting that the CEEC-based protocol can accurately reconstruct the diets of copepods without interference of copepods and their associated ciliates present in the DNA samples. Our initial application to analyzing the food composition of field-collected copepods uncovered diverse prey species, including those currently known, and those that are unsuspected, as copepod prey. While testing is required, this protocol provides a useful strategy for depicting in situ dietary composition of copepods.

## Introduction

As the most numerous animals in marine ecosystem, copepods are critical link of primary production to higher trophic levels, and important driver of the marine biological pump [1]. Copepods can be herbivores, carnivores and omnivores, or can switch their trophic mode according to the relative abundances of the prey species. They can feed on a variety of prey belonging to diverse taxa and size categories, including phytoplankton, protozoans, eggs and larva of aquatic organisms, including those of copepods, and detritus [2]–[4]. Although copepods demonstrate remarkable versatility in their prey, they also exhibit specific feeding preferences among different prey species based on the traits of prey, such as motility, cell size and shape, nutritional value, dissolved chemical cues, and cell surface properties [5]–[8]. For example, both laboratory and field incubation studies have shown that copepods preferentially graze on ciliates and dinoflagellates when diverse foods are offered because they have higher nutritional quality than other prey species and are rich in polyunsaturated fatty acids (PUFA) and eicosapentaenoic acid (EPA) that influence the growth, survival and fecundity of copepods [9]–[10]. In addition, copepods can also discriminate between individuals of the same species with different properties, including biochemical composition, growth stage and nutritional quality [11].

Current knowledge of copepod feeding is largely derived from incubation experiments, which do not necessarily provide information on true diet composition of copepods at sea. Although natural dietary information can be obtained from gut content analysis of wild-caught copepods, the currently available microscopic technique is not only time-consuming but also challenging because partially digested prey can be extremely difficult to identify. Pigment analysis has also been used to investigate prey diversity, but it is limited to phytoplankton and has low taxon-resolving power [12]. Stable isotope analysis of organic materials and fatty acid analysis are helpful in tracing sources of carbon or nitrogen and can provide information of diet categories or trophic level, but still could not reconstruct species composition of the diet [13]–[14].

Molecular techniques have been widely used for detecting the prey composition of aquatic predators in the natural environment due to its sensitivity, specificity and rapidness [15], among which polymerase chain reaction (PCR) assay has been successfully used to detect the food composition of marine invertebrates, such as crustacean (e.g. amphipods, krill, copepods) and mollusca (e.g. bivalves) [16]–[18]. Although PCR assay has also been explored in copepod grazing research, most of these studies so far have focused on detecting predetermined prey species ingested by copepods [4], [18]–[21] or targeted only one type of prey at a time [22], which still cannot provide a whole picture of in situ dietary composition of copepods.

18S ribosomal RNA gene (18S rDNA) is widely used in PCR as a highly sensitive gene marker because it consists of multiple copies in the genomes of eukaryotic organisms [23]. Universal 18S rDNA primers (e.g. 18ScomF1/18ScomR1) have been proven useful in amplifying nearly all of the eukaryotes [24]; however, their application to analyses of food composition in a predator is constrained by the inevitable concurrent amplification of predator 18S rDNA. In our own experiments using DNA from whole copepods (without dissecting guts) as template and 18ScomF1/18ScomR1 primer set for PCR amplification, the clone libraries were typically overwhelmed by 18S rDNA of the copepods. We recently designed a pair of copepod-excluding universal 18S rDNA primers (18S Non-copepod F2R2), and our initial test showed effective depression of copepod 18S rDNA and revealed several symbiotic ciliates associated with various species of copepods [25]. However, the efficiency of the primers to detect a broad range of eukaryotes has not been demonstrated. Besides, the consistent amplification of 18S rDNA from the diverse symbiotic ciliates associated with copepods casts some problems about the applicability of this primer set aimed at recovering all diet species of copepods. In this study, we designed a blocking primer which excludes sequences from those symbiotic ciliates, and combined it with 18S Non-copepod F2R2 to yield a copepod/symbiotic ciliate-excluding, eukaryote-common primer set (18S Non-copepod F2R2+ blocking primer, named as CEEC). We firstly examined the specificity and efficacy of the CEEC primer set by testing 16 copepod species, 30 algal species from 6 phyla, 7 other species from 4 eukaryotic lineages, and a field-collected microplankton sample, and then tested the applicability of the primer set to reconstructing diet composition for both lab-reared and field-collected copepods.

## Materials and Methods

### Algal cultures

Algal cultures were either isolated from South China Sea or obtained from the National Center of Marine Algae and Microbes (NCMA; formerly Provasoli-Guillard Center for the Culture of Marine Phytoplankton or CCMP), Bigelow Laboratory for Ocean Science, Maine, USA. Algae were cultured in f/2 or f/2-Si medium (pH 8.0, salinity 33±1‰) at 20°C on a 14∶10 h light:dark cycle at ∼100 µmol photons·m^−2^·s^−1^ (Table S1).

For each species, about 10,000 cells were harvested during the exponential growth phase by gentle filtration (<50 mm Hg) onto 0.45/3-µm polycarbonate membrane, and then immersed in 0.5 mL DNA lysis buffer (10 mM Tris-HCl pH 8.0, 0.5% SDS, 100 mM EDTA pH 8.0, 200 µg mL^−1^ proteinase K) for subsequent DNA extraction.

### Individual species of other eukaryotes

Seven other eukaryotic species were obtained from field seawater/aquaculture farms of Daya Bay (22°34.76′N, 114°31.52′E) or coral ecosystem in Sanya Bay (18°12.996′N, 109°27.003′E) (Table S1). Individuals or tissues from these eukaryotes were thoroughly ground on liquid nitrogen and then suspended in 0.5 mL lysis buffer for DNA extraction.

### Field-collected natural microplankton samples

For field microplankton samples, 500 mL seawater was collected from 1–2 m below the surface in the Pearl River Estuary (22°7.022′N, 113°52.175′E) using a Niskin bottle (Table 1) and were fixed on site in neutral Utermöhl’s solution [26] at 2% final concentration for later analysis of ambient phytoplankton communities, a method proven to keep DNA intact for several months at room temperature [27]. Upon arrival at the laboratory, 50 mL subsamples were taken and settled down for two days in dark, and then concentrated to 1 mL which were then identified and enumerated in a Sedgwick-Rafter counting chamber under an Olympus BX51 microscope. Another 200 mL subsamples were taken and centrifuged at 3000×g for 15 min, and the concentrated samples were pelleted by centrifugation at 12000×g for 5 min. The cell pellets were re-suspended in 0.5 mL DNA buffer for DNA extraction.

**Table 1:** Information on collection of field samples for different purposes.

Station*	Sampling date	Type	Pre-treatment	Purpose
AV	31-Oct-2007	Live copepods	Gut evacuation and fixed	Primer test
DYW	25-Apr-2011	Live copepods	Gut evacuation and fixed	Primer test
DYW	28-Jul-2012	Live copepods	Gut evacuation and fixed	Primer test
SYB	20-Apr-2010	Live copepods	Gut evacuation and fixed	Primer test
PRE	28-Aug-2011	Fixed water sample		Primer test
DYW	28-Jul-2012	Live copepods	Gut evacuation	Feeding experiment
PRE	28-Aug-2011	Fixed copepods		Diets analysis

*: AV, Avery Point (41°18.917′N, 72°3.81′W), Connecticut, USA; DYB, Daya Bay (22°36.274′N, 114°34.0′E), South China Sea, China; SYB, Sanya Bay (18°12.794′N, 114°34.0′E), South China Sea, China; PRE, Pearl river estuary (22°7.022′N, 113°52.175′E), South China Sea, China.

### Starved copepods

To verify the non-amplification of 18S rDNA from copepods by CEEC primers, live copepods were collected using a zooplankton net (200-µm mesh) from the Avery Point campus of University of Connecticut in Long Island Sound, Pearl river estuary, Sanya Bay and Daya Bay in South China Sea (Table 1). No specific permits were required for the described field studies. A total of 16 species from 10 genera were obtained (Table 2). They were kept alive in filtered seawater during transportation to the laboratory and were starved in the laboratory to evacuate the gut contents, then identified and sorted under a stereoscope. The adult female individuals of the dominant species, *Acartia erythraea*, were sorted out for later feeding experiment (next section). Other copepods were homogenized for DNA extraction as reported [25].

**Table 2:** Copepod species employed for the test of CEEC primer set*.

Genus	Starved copepods	Size (mm)	Gender*	Nutrition mode	PCR results by 18S universal primer set	PCR results by CEEC primer set	Source*
***Acartia***	*A. erythraea*	∼1.45	F	Omnivores	+	−	DYB
	*A*. *pacifica*	∼1.3	F	Omnivores	+	−	DYB
	*A*. *tonsa*	∼1.2	F	Omnivores	+	−	AV
	*A*. *spinicauda*	∼1.4	F	Omnivores	+	−	PRE
***Subeucalanus***	*S. subcrassus*	∼3.0	F	Carnivores	+	−	SYB
***Canthocalanus***	*Can. pauper*	∼1.5	M	Omnivores	+	−	PRE
***Acrocalanus***	*Acr. gibber*	∼1.1	F	Herbivores	+	−	DYB
***Temora***	*T. turbinata*	∼1.35	F	Herbivores	+	−	SYB
	*T*. *discaudata*	∼1.8	F	Herbivores	+	−	SYB
***Pontellopsis***	*P. tenuicauda*	1.65	F	Carnivores	+	−	SYB
***Centropages***	*C. tenuiremis*	∼1.5	F	Omnivores	+	−	DYB
	*C*. *furcatus*	1.65	F	Omnivores	+	−	SYB
***Tortanus***	*T. gracilis*	1.75	F	Omnivores	+	−	SYB
***Paracalanus***	*Par. parvus*	∼0.8	F	Herbivores	+	−	DYB
***Calanopia***	*Cal. thompsoni*	∼1.85	M	Omnivores	+	−	SYB
	*C*. *elliptica*	∼1.75	M	Omnivores	+	−	SYB

*Adult copepods were used in this experiment, F: female, M: male. The symbol “+” denotes positive result; “−” denotes negative result; * DYB, Daya Bay (22°36.274′N, 114°34.0′E), South China Sea, China; AV, Avery Point (41°18.917′N, 72°3.81′W), Connecticut, USA; PRE, Pearl river estuary (22°7.022′N, 113°52.175′E), South China Sea, China; SYB, Sanya Bay (18°12.794′N, 114°34.0′E), South China Sea, China.

### Copepods fed on artificially mixed diet

To test whether the primer set could efficiently amplify individual species in a mixed diet, a short-time laboratory feeding experiment was carried out. Starved copepods (*A. erythraea*) were introduced into the feeding chamber at 3.75 ind·mL^−1^ and fed with a mixture of different algal species with equal biovolume basis (∼1.0×10^8^ µm^3^), including *Prorocentrum donghaiense, Thalassiosira weissflogii, Phaeodactylum tricornutum* and *Tetraselmis suecica*. Every 3 minutes, copepod samples were taken and checked under a stereoscope to determine the feeding condition. Once the guts of copepods were filled and no feces were detected (<15 min), samples were collected gently and rinsed carefully with filtered seawater, and then preserved for DNA extraction.

### Field-collected copepod samples

Field copepod samples were also collected from the same sampling site in Pearl River Estuary waters (22°7.022′N, 113°52.175′E) using a 505-µm zooplankton net (Table 1). Once retrieved from the cod end, the sample was preserved immediately using neutral Utermöhl’s solution and transferred to laboratory where the dominated species, *Canthocalanus pauper*, was sorted out for DNA extraction.

### DNA extraction

Algal and microzooplankton cell pellets and copepod homogenates were incubated in lysis buffer for three days at 55°C for thorough cell lysis, and DNA was extracted following a modified CTAB protocol, and finally eluted in 50 µL 10 mM Tris-HCl (pH 8.0), as previously reported [24].

### PCR protocol

Due to the previously reported prevalent association of symbiotic ciliate with copepods [25], we developed a blocking primer (ciliate18Sblk2: 5′-CCCAATCCTGATCCAGGGAGGTAGTGACAAGAAATAACAACCTAGTCGCAAGACACACC-3′) to specifically depress the amplification of 18S rDNA from those ciliates. Combined primers Non-copepod 18SF2, ciliate18Sblk2 and Non-copepod18SR2 were used in a ratio of 1∶4∶1. DNA from 37 eukaryotic organism, 16 copepod species, and a natural microplankton sample were PCR-amplified using this primer set under the same program as previously reported [25]. PCR products were purified and cloned into T-vector, and 50 to >100 clones were sequenced as reported [24]. To exclude the possibility that the negative result of the copepods DNAs was due to poor quality of the extracted DNA, each DNA sample was also PCR-amplified using the universal 18S rDNA primer set under the PCR conditions as reported previously [24].

### Sequence data analysis and phylogenetic inference

Sequences obtained were searched against the GenBank database using the Basic Local Alignment Search Tool. The best hits were collected and aligned with the new sequences obtained in this study using CLUSTAL W (1.8) [28]. Maximum Likelihood (ML) tree was inferred from the aligned dataset.

## Results

### Efficacy of CEEC primers to depress amplification of copepod 18S rDNA

PCR with CEEC primer set on the DNA from 16 starved copepod species all gave negative results (Table 2). However, PCR with the universal 18S rDNA primer set all produced an amplicon with an expected product of ∼1.8 kb, indicating that the negative results with CEEC primers were not due to poor DNA quality, and cloning and sequencing results showed that they were all copepods or symbiotic ciliates. These results thus indicated that the primer set could effectively avoid amplification of 18S rDNA from the common copepod species and successfully block symbiotic ciliates sequences.

### PCR efficacy in detecting individual eukaryotic species

**Figure 1:**
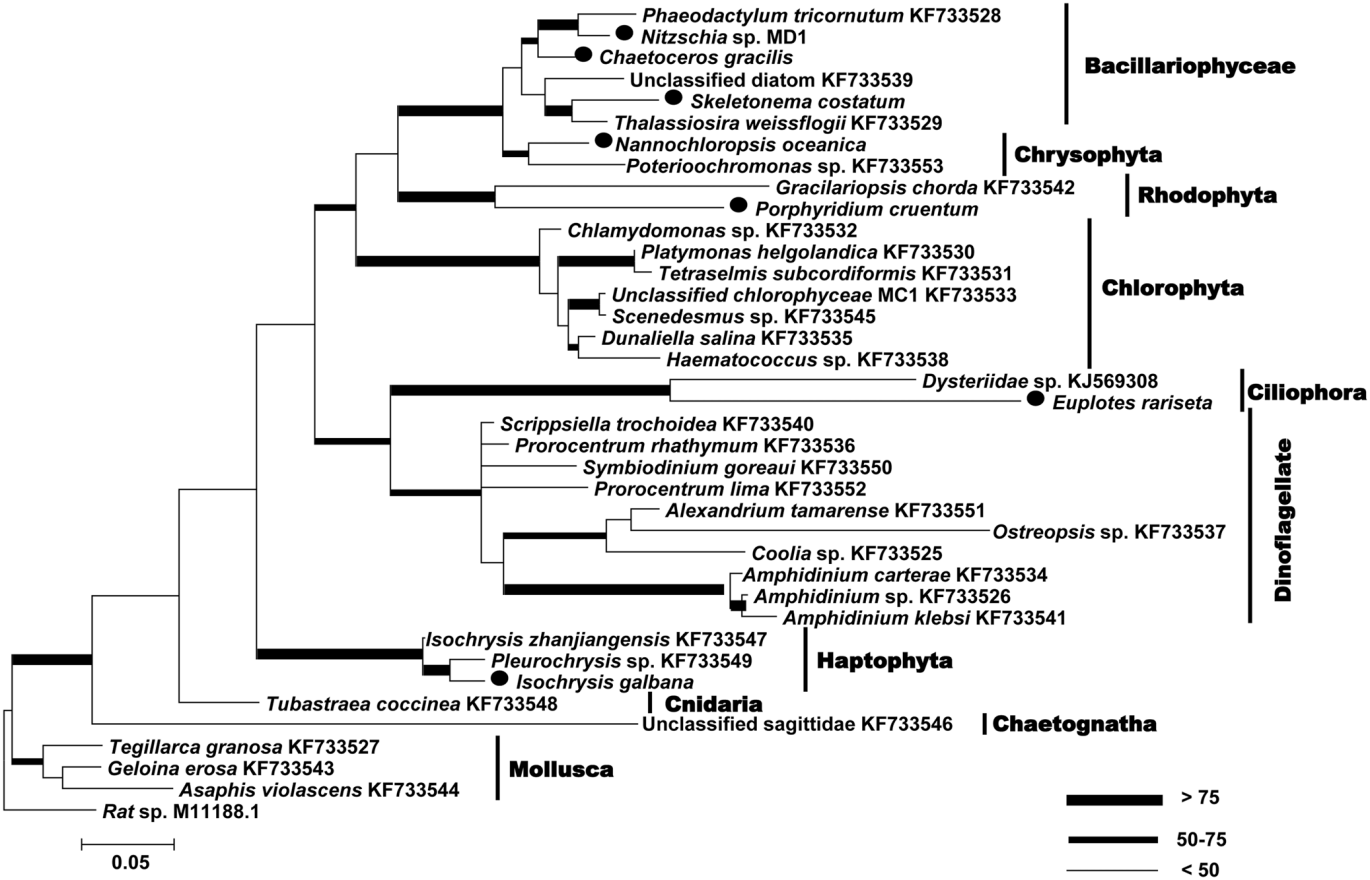
Maximum Likelihood (ML) phylogenetic tree of 18S rDNA gene from different eukaryotes amplified by CEEC primer set. Sequences marked with solid circles are 100% identical to those under GenBank accession numbers AY229897.1, HM805045.1, JQ315726.1, HM149540.1, GQ246179.1, HM246242.1 and AJ305248.1. Other sequences obtained for mono-species were all submitted to GenBank and the accession numbers were shown in the tree (KF733525–KF733553, KJ569308).

The efficacy of CEEC primer set was confirmed by the positive PCR results from all 37 different species examined (Table S1), which represented a wide phylogenetic range of eukaryotes (Figure 1). Sequences obtained for *Chaetoceros gracilis*, *Skeletonema marinoi (costatum)*, *Nannochloris oculata*, *Isochrysis galbana* and *Nitzschia* sp. (MD1) were 100% identical to the sequences from corresponding species documented in GenBank (AY229897.1, HM805045.1, JQ315726.1, HM149540.1 and GQ246179.1, respectively). Sequences from dinoflagellates *Coolia* sp., *Amphidinium* sp. and *Prorocentrum* sp., which were not morphologically identified to species, gave 98–99% identity to sequences from the respective genera in GenBank. All other algal sequences showed 99% identity to those from different strains of the same genus or family. Sequences from ciliate *Dysteriidae* sp. and *Euplotes* sp. showed 99% and 100% identity to a cyrtophorid ciliate *Dysteriidae* sp. (KF384515.1) and *Euplotes rariseta* (AJ305248.1), respectively. Sequences from the other metazoan species all gave 98–99% identity to those from corresponding species documented in the database.

### PCR efficacy in revealing natural microplankton community

Microscopic observation of water sample showed 28 different species, including 19 diatoms, 8 dinoflagellates and 1 metazoan species (Appendicularia: O*ikopleura* sp.). Of the dominant diatom (70%), *Rhizosolenia* showed the highest diversity, with 7 species in this genus, followed by *Coscinodiscus*, which contained 3 species. *Gymnodiniaceae* and *Prorocentraceae* dominated the dinoflagellate community (Table 3). Concerning the cell abundance, diatoms dominated the plankton community (98%), among which *Rhizosolenia* sp. was the most abundant species and accounted for about 57% of total abundance, followed by *Thalassiosira* sp. with 15%.

**Table 3:** Species composition of water sample by microscopic and molecular analyses.

Genus	Microscopic analysis				Molecular analysis	
	Cell density (cells/L)	Percentage of abundance (%)	Species number	Species percentage (%)	Clone number	Percentage (%)
**Diatoms**						
*Rhizosolenia*	1.48×10^6^	56.68	7	25.93	10	13.17
*Thalassiosira*	3.88×10^5^	14.90	2	7.41	7	9.20
*Coscinodiscus*	2.4×10^4^	0.92	3	11.11	3	3.95
*Biddulphia*	1.2×10^4^	0.46	2	7.41	2	2.63
*Skeletonema*	1.64×10^5^	6.30	2	7.41	2	2.63
*Nitzschia*	4.84×10^5^	18.59	1	3.70	4	5.26
*Navicula*	8.0×10^3^	0.31	2	7.41	–	–
Unclassified	–	–			8	10.53
**Dinoflagellates**						
*Gymnodiniaceae*	1.6×10^4^	0.61	2	7.41	6	7.90
*Prorocentraceae*	1.2×10^4^	0.46	2	7.40	2	2.63
*Ceratium*	8.0×10^3^	0.31	1	3.70	–	–
*Peridiniaceae*	8.0×10^3^	0.31	2	7.41	1	1.32
*Heterocapsa*	4.0×10^3^	0.15	1	3.70	–	–
*Syndiniales*		–			7	9.20
**Nanoflagellates**		–			2	2.63
**Cryptophyta**		–			1	1.32
**Chlorophyta**		–			1	1.32
**Chrysophyta**		–			2	2.63
**Metazoan**	35(ind/L)				18	23.68
**SUM**		100			76	100

**Figure 2:**
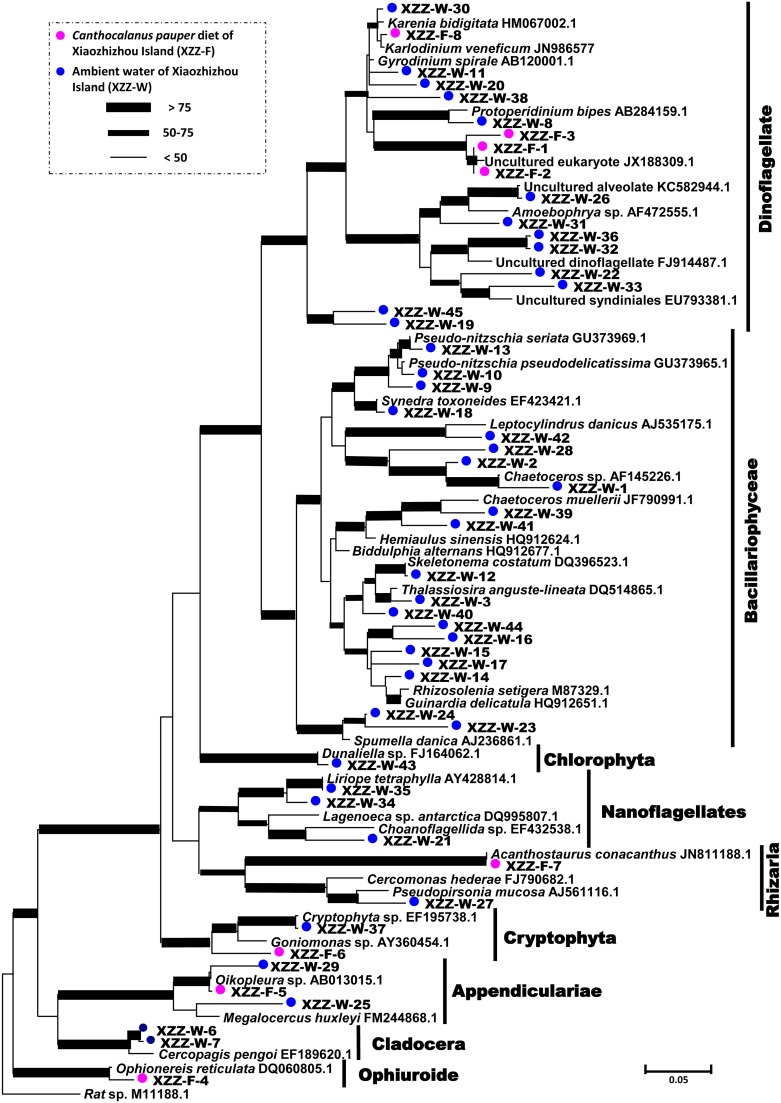
18S rDNA phylogram for grazer *Canthocalanus pauper* and ambient plankton community in Pear River Estuary. Maximum Likelihood (ML) tree was shown here and only representative clones from each major lineage were included in the tree. The color circles denote the sources of the clones. XZZ-W, ambient water sample; XZZ-F, *Can. pauper* sample.

Seventy six clones of 18S rDNA PCR product were sequenced for the water sample, of which 35 genotypes were resulted (Figure 2), and as BLAST analysis showed, these comprised 18 species of diatoms, 9 species of dinoflagellates, 1 species of Cryptophyta, 1 species of Chlorophyta, 1 species of Chrysophyta, 1 species of Cladocera, 2 species of Appendicularia and 2 unclassified nanoflagellates. Sequences from diatom dominated the clone library (47% by clone numbers), with *Rhizosolenia* sp. being the most abundant species. For the phytoplankton community, diatom species from *Rhizosolenia*, *Thalassiosira*, *Coscinodiscus*, *Biddulphia*, *Skeletonema* and *Nitzschia*, and dinoflagellates from *Gymnodiniaceae* and *Prorocentraceae*, were both detected in microscopic results and molecular detection. Compared to the microscopic results, a higher diversity of species was detected by the PCR assay (Table 3). These indicated that CEEC primers can successfully be used in amplification of DNA from assemblage of different organisms except those from copepods and symbiotic ciliates.

### PCR efficacy in reconstructing diet composition of experimentally fed copepods

BLAST analysis of the sequences obtained from lab-fed copepods against the GenBank database showed that they were highly similar (>99% identity) to sequences from *T. weissflogii* (GU594641.1), *P. tricornutum* (FR744760.1), *Pro. donghaiense* (DQ336054.1) and *T. suecica* (FJ559381.1) in GenBank. This result indicated that the primer set could amplify all the mixed prey species in the guts of the laboratory fed copepods.

### Analysis of in situ copepod diets

**Figure 3:**
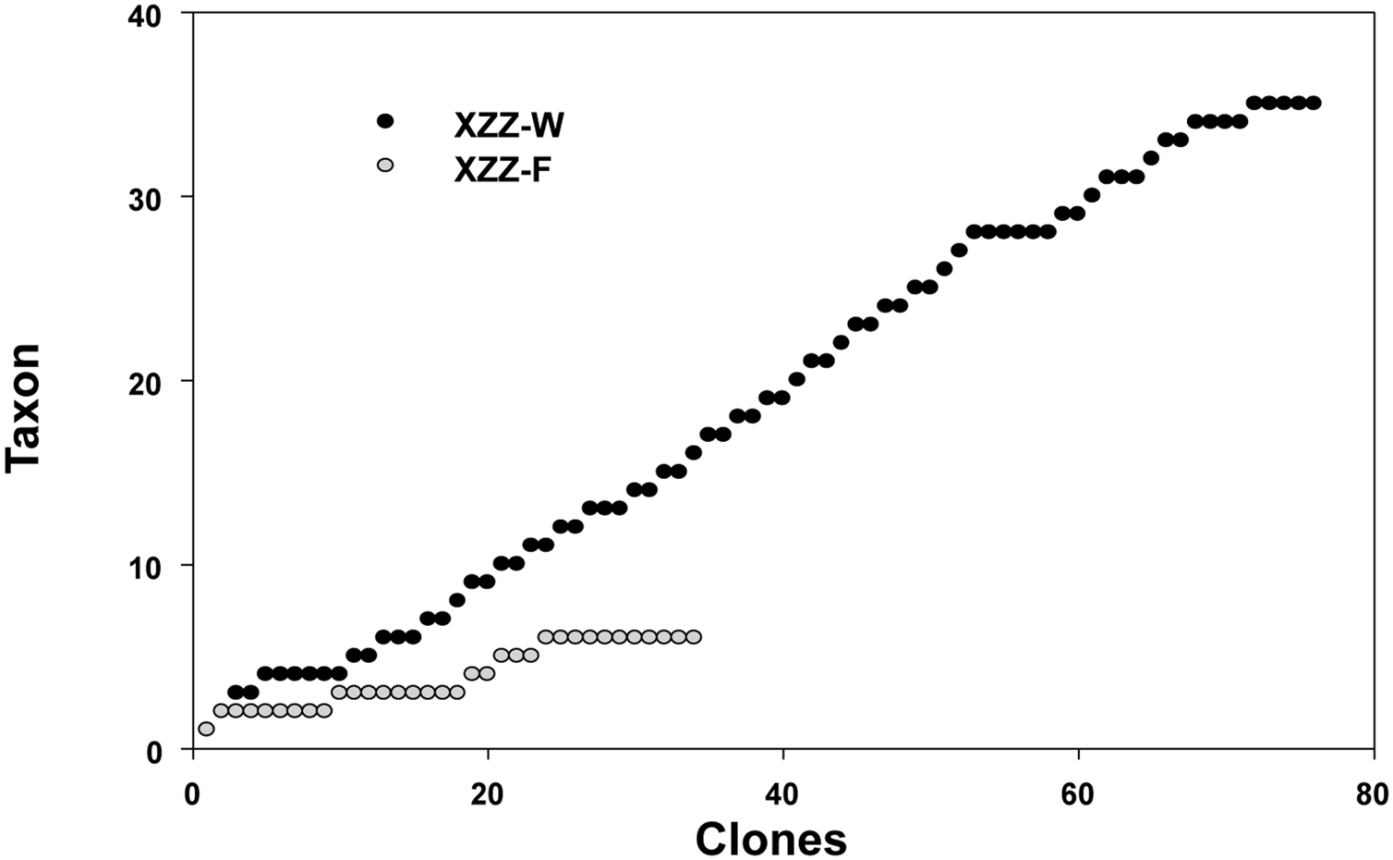
Rarefaction curves for water sample and *Canthocalanus pauper* clone libraries. The asymptote reached in XZZ-F suggests that the numbers of unique taxa in diets of *Can. pauper* was about 6, and no plateau reached for the XZZ-W plot indicates potentially higher diversity in water sample.

For the field-caught copepod (*Can. pauper*) samples, 34 sequences were obtained, of which 6 taxa were identified (Figure 3), including those that were related to dinoflagellates, cryptophyte, radiolaria, echinoderm and tunicate (Table 4, Figure 2). Dinoflagellates were the dominant species in the copepod gut with one sequence showing high similarity to *Karlodinium veneficum* (JN986577, 99% identity) and most of other sequences relatively closely (90%–93% identity) related to documented *Karenia mikimotoi* (FR865627.1). The other 5 sequences were close to that of the brittle star *Ophionereis reticulata* (DQ060805, 97% identity), the tunicate *Oikopleura* sp. (AB013015, 99% identity), the cryptophyte *Goniomonas* sp. (AY360454, 91% identity) and the radiolarian *Acanthostaurus purpurascens* (JN811224, 99% identity), respectively.

**Table 4:** Taxonomic distribution of 18S rDNA clones retrieved from *Can. Pauper.*

Clone ID	Best hit species	Best hit Accession No.	E-value	Identities	Clone number	Class
XZZ-F-1	*Karenia mikimoto*	FR865627.1	0	90%–93%	29	Dinophyceae
XZZ-F-4	*Ophionereis reticulata*	DQ060805	0	97%	1	Echinoderm
XZZ-F-5	*Oikopleura* sp.	AB013015	0	99%	1	Appendicularia
XZZ-F-6	*Goniomonas* sp. ATCC 50108	AY360454.1	0	91%	1	Cryptophyte
XZZ-F-7	*Acanthostaurus purpurascens*	JN811224	0	99%	1	Radiolaria
XZZ-F-8	*Karlodinium veneficum*	JN986577	0	99%	1	Dinophyceae
SUM					34	

## Discussion

Molecular techniques have the advantages for reconstructing diet composition of natural population of copepods and other zooplankton in that they can detect partially digested, morphologically indistinguishable, and low-abundance species. Yet their applications have been restricted by the lack of broad-target DNA markers [17]. The present study was an attempt to develop and validate a copepods/symbiotic ciliates-excluding universal 18S rDNA-based PCR assay. We systematically evaluated the utility of this PCR assay for detecting diverse diets of copepods in natural environments by testing it against individual species of eukaryotic organisms, mixed species, natural assemblage, laboratory fed copepods, and field-caught copepods. The results indicate that this PCR assay largely meets the requirement of an easily accessible, universal, and sensitive method.

One potential major issue of applying such a PCR assay to detect diet diversity in copepods is the interference of copepod DNA, which is usually dominant in the DNA sample extracted from whole copepods [29]. Although isolating guts for DNA extraction can reduce this problem, it is time-consuming and still cannot guarantee complete removal of gut tissue-derived copepod DNA. In the present study, we showed that use of CEEC primers was very helpful in depressing amplification of copepod 18S rDNA for all copepods we examined. Most of the copepods tested were dominant species of coastal ecosystem from temperate or subtropical/tropical regions, such as *A. tonsa* in coastal of Atlantic Ocean [30] and *A. erythraea* in South China Sea [31]. These copepods had different individual size, from<1 µm (such as *P. parvus*) to>3 µm (such as *S. subcrassus*) [31] and exhibited different feeding habits (herbivorous, carnivorous and omnivorous) [32]. Therefore, the CEEC primer set can be used to analyze diets for these, and likely other, copepods in various natural marine environment in future. The method will be particularly useful for small copepods, whose guts are very difficult to dissect for prey identification and feeding ecology is less understood.

For a PCR assay to be useful in detecting food diversity, a wide taxonomic coverage is needed. In the present study, we used the CEEC primer set to amplify 37 different eukaryotic species. Most of these were phytoplankton, which is considered as main diet components of mesozooplankton [22]. Because previous microscopic studies implied that the herbivores and omnivorous copepods might feed on unidentified diet materials derived from flagellates, aloricate ciliates, athecate, dinoflagellates or detritus [33], we also tested 2 species of free-living ciliates, which had been shown to be important prey item for copepods in laboratory experiments, and 5 field-collected marine metazoan, whose eggs, larvae or body parts might be ingested by copepods [3]. All the above phytoplankton and other eukaryotic species were successfully amplified, which were verified by sequencing results, indicating that the PCR assay is efficient in detecting a wide range of different mono eukaryotic species. In addition, a natural microplankton sample from the ambient water was chosen to evaluate whether the primer set can detect diverse species from a mixed sample. As the clone and sequencing results showed, much higher diversity of eukaryotic species (more than 35 species) was detected than that revealed by microscopic identification (28 species). Almost all the phytoplankton groups revealed by microscopic identification were present in the clone library except *Ceratium* and *Heterocapsa*, which were missed likely due to insufficient sequencing depth, as suggested by the still growing taxon number in the rarefaction curve (Figure 3). It is noteworthy that nanoflagellates and cryptophyta, which might be difficult to identify under light microscope, were detected, indicating its higher sensitivity to allow detection of small-sized and low-abundance taxa [30]. Taken together, all our results show that the primer set will be useful in revealing in situ copepod diet diversity.

The next challenge is to verify that the PCR assay would be sensitive and specific enough to detect prey in the gut when DNA from whole copepods is used. When our PCR protocol was applied to detect prey species in laboratory-fed copepods, all the prey species offered were successfully amplified. In the application to field-collected copepods, diverse and some unsuspected species in the diets were detected. Our results showed that the diet of *Can. pauper* ranged from phytoplankton to metazoan, which is consistent with the previous report that *Can. pauper* is omnivorous [2]. It is reported that dinofalgellates contribute a large portion of the carbon in the diets of common estuarine species [34]. In accordance, with the protocol developed here, we found many dinoflagellate 18S rDNA sequences. As some species of cryptophyte (such as *Rhodomonas* sp.) has been considered to be prey of copepods, we also detected cryptophyte although the species (*Goniomonas* sp.) has not been reported [19]. Skeletal remnants of radiolarians have also been found in the guts of large copepods (e.g. *Pleuromamma xiphias* and *Euchirella messinensis*) by gut contents analysis in Sargasso Sea [3]. As gut dissection for small copepods, such as *Can. pauper* studied here, is difficult, metazoans as prey for copepods were rarely reported. Furthermore, brittle star and tunicate have not been reported as prey of copepods, but retrieved from field-caught copepods in this study. Therefore, the PCR protocol developed in this study has higher sensitivity and resolution in detecting in situ food composition of copepods.

Besides excluding copepods, our PCR protocol was also designed to exclude epibiotic apostomatid ciliates (mainly *Vampyrophrya-*like and *Vorticella-*like species) by using a blocking primer. However, it is important to verify that the protocol would not block out other ciliates that might be food of copepods. Two free-living ciliates, which are considered to be important prey item for copepods [2], could be successfully amplified by the primer set. Similarly, even though the protocol was intended to exclude grazer copepods, we did not want to also exclude other crustaceans. From the ambient water sample, we detected Cladocera (95–96% identical to *Cercopagis pengoi*, EF189620.1), which accounted for 17% of total clones. Furthermore, some other species (such as *Farfantepenaeus duorarum*) were also detected from copepod samples (unpublished data), indicating that the primer set could specifically evade amplification of copepod but other crustacean, larvae or body parts of which might be potential preys of some copepods [3].

Compared with pigment analysis or gut contents microscopy, this method can reveal a higher diversity of diets for copepods without gut dissection, which could help reconstruct copepod feeding conditions in natural waters. As demonstrated by previous studies that copepods can graze on other copepods or even their own nauplii to meet their metabolic costs in field, the PCR assay developed in this study might cause underestimation of diet diversity as designed to exclude copepods and thus would miss eggs, larvae, or adults of these copepods that might be a prey [2], [16]. Possible retrieval of the missing information would rely on species-specific PCR assays requiring prior knowledge about what species may be a prey. For instance, Durbin et al. (2008) reported that adult female *Centropages typicus* can feed on nauplii of *A. tonsa* by using species-specific primers for the mitochondrial cytochrome oxidase subunit one gene (mtCOI) [35].

As the first attempt of its kind, the evaluation results of the copepod-excluding PCR assay indicate that it is a promising protocol to allow us to detect non-copepod-prey diversity of a broad range of copepod species, information very useful for understanding trophic networks in the natural marine environment [22]. It can be envisioned that the application of this protocol, in conjunction with some of the existing methods, to field copepod populations will bring to light many unrecognized trophic relationships in the vast marine ecosystem, although the applicability of the protocol for quantitative analysis of grazing on each prey species has yet to be investigated.

## Supporting Information

Table S1 **Individual marine eukaryotes used for primer test.**
(DOCX)
