# Supplementary material for: Detecting In Situ Copepod Diet Diversity Using Molecular Technique: Development of a Copepod/Symbiotic Ciliate-Excluding Eukaryote-Inclusive PCR Protocol
Source: PLoS One. 2014 Jul 24;9(7):e103528. doi: 10.1371/journal.pone.0103528 (PMC4110036; doi:10.1371/journal.pone.0103528)
Supplement: Table S1 — Individual marine eukaryotes used for primer test. (DOCX) [file pone.0103528.s001.docx]

Table S1. Individual marine eukaryotes used for primer test

| **Lineage** | **Species** | **Medium** | **Type** | **Source** |
| --- | --- | --- | --- | --- |
| **Bacillariophyta** | *Skeletonema marinoi (costatum)* | f/2 | Cultured | South China Sea |
|  | *Thalassiosira weissflogii* | f/2 | Cultured | CCMP 1048 |
|  | *Phaeodactylum tricornutum* | f/2 | Cultured | CCMP 630 |
|  | *Nitzschia* sp. (MD1) | f/2 | Cultured | South China Sea |
|  | *Chaetoceros gracilis* | f/2 | Cultured | South China Sea |
|  | Unclassified diatom | f/2 | Cultured | South China Sea |
| **Chrysophyta** | *Nannochloropsis oceanica* | f/2-Si | Cultured | South China Sea |
|  | *Poterioochromonas* sp. | f/2-Si | Cultured | South China Sea |
| **Haptophyceae** | *Phaeocystis* sp. | f/2-Si | Cultured | South China Sea |
|  | *Isochrysis zhanjiangensis* | f/2-Si | Cultured | South China Sea |
|  | *Isochrysis galbana* | f/2-Si | Cultured | CCMP 1323 |
| **Pyrrophyta** | *Coolia* sp. | f/2-Si | Cultured | South China Sea |
|  | *Alexandrium catenella* | f/2-Si | Cultured | ACHK |
|  | *Ostreopsis* cf.*ovata* | f/2-Si | Cultured | South China Sea |
|  | *Amphidinium carterae* | f/2-Si | Cultured | South China Sea |
|  | *Amphidinium klebsi* | f/2-Si | Cultured | South China Sea |
|  | *Amphidinium* sp. | f/2-Si | Cultured | South China Sea |
|  | *Symbiodinum goreaui* | f/2-Si | Cultured | South China Sea |
|  | *Prorocentrum lima* | f/2-Si | Cultured | South China Sea |
|  | *Prorocentrum rhathymum* | f/2-Si | Cultured | South China Sea |
|  | *Scrippsiella trochoidea* | f/2-Si | Cultured | South China Sea |
| **Chlorophyta** | *Platymonas helgolandica* | f/2-Si | Cultured | South China Sea |
|  | *Tetraselmis subcordiformis* | f/2-Si | Cultured | South China Sea |
|  | *Chlamydomonas* sp. | f/2-Si | Cultured | South China Sea |
|  | Unclassified chlorophyceae (MC1) | f/2-Si | Cultured | South China Sea |
|  | *Scenedesmus* sp. | f/2-Si | Cultured | South China Sea |
|  | *Dunaliella salina* | f/2-Si | Cultured | CCMP1320 |
|  | *Haematococcus* sp. | f/2-Si | Cultured | South China Sea |
| **Rhodophyta** | *Porphyridium cruentum* | f/2-Si | Cultured | South China Sea |
|  | *Gracilariopsis chorda* | − | Cultured | South China Sea |
| **Ciliophora** | *Dysteriidae* sp. | **−** | Field sample | Daya Bay |
|  | *Euplotes rariseta* | **−** | Field sample | Daya Bay |
| **Cnidaria** | *Tubastraea coccinea* | *−* | Field sample | Sanya Bay |
| **Chaetognatha** | *Sagittidae* sp. | *−* | Field sample | Sanya Bay |
| **Mollusca** | *Tegillarca granosa* | *−* | Field sample | Daya Bay |
|  | *Asaphis violascens* | *−* | Field sample | Daya Bay |
|  | *Geloina erosa* | *−* | Field sample | Daya Bay |
